# Novel Softwood Lignin Esters as Advanced Filler to PLA for 3D Printing

**DOI:** 10.1021/acsomega.4c06680

**Published:** 2024-10-24

**Authors:** Mahendra K. Mohan, Illia Krasnou, Tiit Lukk, Yevgen Karpichev

**Affiliations:** † Department of Chemistry and Biotechnology, Tallinn University of Technology (TalTech), Akadeemia tee 15, 12618 Tallinn, Estonia; ‡ Department of Materials and Environmental Technology, Tallinn University of Technology (TalTech), Ehitajate tee 5, 19086 Tallinn, Estonia

## Abstract

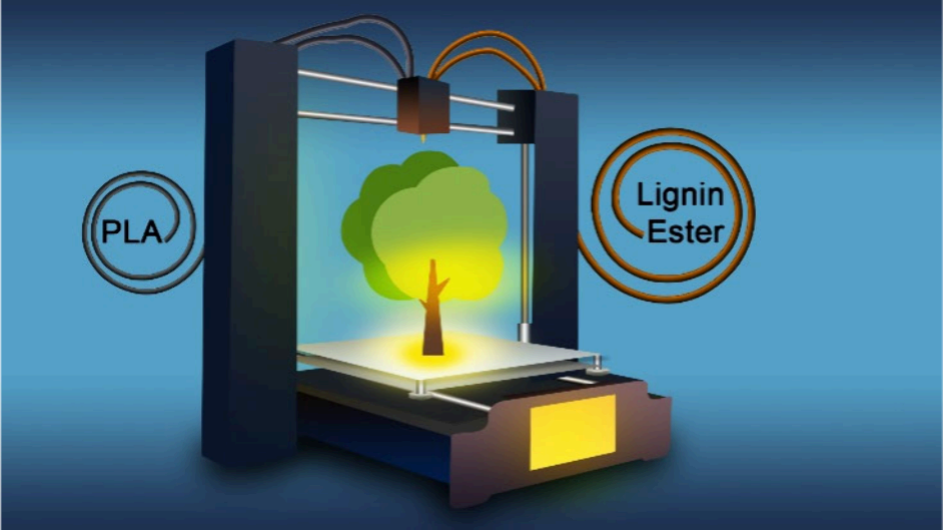

In this study, we explored the selectivity of softwood lignin toward esterification through chloromethylation. Organosolv pine lignin chloromethylated by a novel greener protocol was subjected to esterification with decanoic acid (C10), tetradecanoic acid (C14), and stearic acid (C18). The success of lignin esterification was confirmed by using FTIR and NMR spectroscopy. For composite preparation, modified lignin was incorporated with PLA in varying proportions (10%, 20%, 30%, and 40%) using the solvent casting technique. The thermal and mechanical properties of the solvent-cast films were analyzed. Notably, lignin esters increased the glass transition temperature (*T*_g_) of PLA by a few degrees: tetradecanoic acid (C14) at 30% loading exhibited increases *T*_g_ from approximately 68 to 72 °C. Mechanical testing showed that blending PLA with lignin and its ester derivatives improves its properties. Pure PLA has moderated ductility and stress but lowered stress levels compared to PLA-lignin ester blends. Adding 30% lignin reduced strength and strain, making the material more brittle. In contrast, the PLA + lignin C14 ester (30%) blend achieved the highest stress and strain, enhancing toughness and strength. This makes lignin C14 ester a promising additive for improving the mechanical properties of PLA in applications requiring greater strength and toughness. The composition with optimum properties was selected for production of the 3D-printing filament. Three extrusion temperatures were evaluated, and the advanced mechanical properties of 3D-printed filament along with surface morphology were analyzed.

## Introduction

The Intergovernmental Panel on Climate Change (IPCC) estimates that global temperatures have risen by about 1.2 °C (2.2 °F) since the preindustrial era. This global warming is mainly driven by human activities, such as burning fossil fuels, deforestation, and industrial processes, which have led to unprecedented levels of greenhouse gas emissions. Approximately one-third of global primary energy demand comes from the building sector, making it a significant source of energy-related greenhouse gas (GHG) emissions.^1^ As a result, rapid and extensive climate change has profound impacts on weather patterns, ecosystems, and human societies. Additionally, the manufacturing of construction materials accounts for over 80% of the energy consumption in building construction.^2^

Plastics play a pivotal role in various commercial sectors, contributing to a total production volume of approximately 450 million tons annually in 2019.^3^ However, fossil-based plastics are closely associated significant environmental challenges, including CO_2_ emissions and littering, which contribute to widespread microplastic pollution. As the global population continues to rise, there is an anticipated surge in the demand for plastic products, even as environmental concerns drive the necessity for the development of renewable alternatives.^4^ Presently, the annual production capacity of bioplastics accounts for less than 1% of the total plastic production volume,^5^ underscoring the urgent need for innovative solutions to meet the growing demand for renewable materials and substitute fossil-based plastics.^6^

Increasing evironmental awareness is promoting the adoption of greener and high-thermal-performance sustainable materials for construction. Biobased materials are becoming essential for enhancing the energy efficiency of buildings, offering both environmental and economic advantages.^7^ Utilizing plant-based biomass materials in construction can decrease fossil energy demand, lower carbon dioxide emissions, and reduce the generation of nondegradable waste.

PLA, derived from a renewable agricultural-based monomer, 2-hydroxypropionic acid (lactic acid), is a versatile biopolymer synthesized through the fermentation of starch-rich materials like sugar beets, sugar canes, and corn.^8^ Besides its innate biocompatibility, PLA finds extensive application across diverse fields, including 3D printing^9,10^ and beyond. However, despite its potential, PLA faces inherent limitations such as brittleness, low heat resistance, high cost and slow crystallization, which have hindered its widespread adoption in commercial applications.^11^ Also, it is relatively expensive and produced from starch, which competes with the food supply chain. Thus, the development of sustainable nonfood-based bioadditives for plastics is an approach worthy of further development.

Lignin, an amorphous polyphenol present in plant cell walls, possesses a random, three-dimensional structure formed through an enzyme-mediated dehydrogenative polymerization of phenyl propanoic precursors such as coniferyl, sinapyl, and *p*-coumaryl alcohols. Softwood trees typically contain approximately 28% of lignin, predominantly composed of over 95% guaiacyl units (4-hydroxy-3-methoxy) and traces of *p*-hydroxyphenyl units.^12^ It has been clearly observed that the glass transition temperature (*T*_g_) of softwood lignin was much higher than that of hardwood lignin.^13^ There is increasing interest in leveraging lignin as an affordable and ecofriendly raw material to enhance its utility and broaden its applications. Additionally, there is renewed attention toward producing green and cost-effective polymer composites, where lignin can be incorporated into thermoplastics such as polylactic acid (PLA).^14^

Esterification, which is one of the simplest chemical reactions due to its reaction parameters and reactants, enables the modification of certain properties of lignin.^15^ This includes enhancing its hydrophobicity and solubility in organic solvents.^16^ Furthermore, esterification functionalizes hydroxyl groups on lignin with ester substituents,^17^ reducing hydrogen bonding and increasing molecular free volume.^18^ Consequently, this enhances chain mobility and lowers the glass transition point of lignin, thereby increasing its thermoplasticity.^19^

This study aims to incorporate organosolv pine lignin, chemically modified via a greener chloromethylation protocol (reported by our team recently^20,21^) followed by esterification, into PLA as a high-performance filler capable of not only decreasing the amount of PLA in filament targeting a lower price of the final material but also enhancing its thermal and mechanical properties. Given our understanding that unmodified lignin alone does not enhance thermal properties, we chose to conduct a simple two-step esterification process using decanoic, tetradecanoic, and stearic acids. This process aimed to chemically modify lignin obtained from chloromethylated lignin while preserving the hydroxyl groups of lignin. In this research, we synthesized a range of lignin/PLA composites using a solvent casting technique. It is anticipated that the aliphatic groups of long-chain fatty acid esters present on the surface of lignin would enhance adhesion to the PLA matrix during processing. Additionally, the unmodified hydroxyl group on lignin is expected to elevate intermolecular forces, interchain attraction, and cohesion, consequently raising *T*_g_ by reducing mobility. Hence, this study aims to assess the thermal, mechanical, and morphological properties of the resulting lignin-based PLA biocomposites, demonstrating their suitability as filament materials for 3D printing.

## Experimental Section

### Materials and Methods

#### Chemicals

All reagents utilized, of analytical reagent (AR) grade and procured from Sigma-Aldrich (Taufkirchen, Germany), were employed without further purification. PLA-Ingeo 3D850 was obtained from NatureWorks LLC, US. Deionized water obtained from a Milli-Q water purification system (Millipore S. A., Molsheim, France) was utilized throughout the study. Longitudinally sawn pine timber sawdust was sourced from Prof. Jaan Kers (Tallinn University of Technology, Tallinn, Estonia). The feedstocks underwent drying in a convection oven at 50 °C until reaching 8% moisture content, followed by grinding to a fine powder and subsequent storage in plastic bags at room temperature.

#### Methods

The FT-IR analysis was performed with a Shimadzu IRTracer-100 spectrometer (Kyoto, Japan) in attenuated total reflection (ATR) mode, featuring a resolution of 2 cm^–1^ and 80 scans. Shimadzu Lab Solutions software was used to analyze all samples within the 400–4000 cm^–1^ range. For ^1^H NMR analysis, approximately 40 mg of each sample was dissolved in DMSO-*d*_6_ or CDCl_3_ and placed in a 5 mm NMR tube. The spectra were recorded using a Bruker Avance III 400 MHz spectrometer, and the ^1^H spectra were analyzed with MestReNova x64 software.

#### Extraction and Chloromethylation of Lignin

**Scheme 1:**
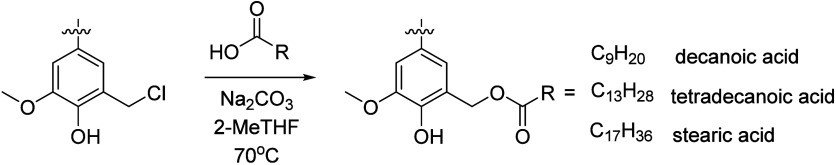
Synthesis Pathway of Lignin Esterification

Lignin was extracted from pine according to the previously described organosolv procedure,^22^ and dried organosolv lignin was then weighed (yield 6%) and used for either subsequent analysis or following procedures. Chloromethylation of organosolv lignin was performed according to our previously described procedure^20^ (see Scheme 1).

#### Esterification of Organosolv Pine

For esterification of chloromethylated lignin (see Scheme 1), 1 g (1 equiv) of acids (decanoic acid, tetradecanoic acid, and stearic acid) and 1.2 equiv of Na_2_CO_3_ (0.732, 0.552, 0.443 g) were added to 2-MeTHF (10 mL). The mixture was then heated to 70 °C and maintained at this temperature for 30 min. Subsequently, 1 g of dissolved chloromethylated lignin in 10 mL of 2-MeTHF was added to the reaction mixture, which was then stirred overnight at 70 °C. Upon completion of the reaction, the mixture was poured into cold brine, resulting in the formation of a brown precipitate. The precipitate was collected via filtration and washed with water to remove the salts. Finally, the brown precipitate was dried under a vacuum for subsequent steps.

#### PLA/Lignin Film Preparation

PLA was individually dissolved in DCM within a 15 mL glass vial at 45 °C. Lignin was dissolved in THF at room temperature and subsequently mixed to have a content of 10–40% w/w, according to Table S1. The dissolved components were then combined in a Petri dish and left to dry overnight. Following this initial drying period, the composite material underwent further drying in a vacuum oven at 40 °C for an additional 12 h.

#### Differential Scanning Calorimetry

Differential scanning calorimetry (DSC) was performed by a PerkinElmer Diamond DSC calorimeter (USA) by heating from 0 to 250 °C at a rate of 20 °C/min in a nitrogen atmosphere (purge at 20 mL/min) and then cooling at the same rate. Samples of 4.00 ± 0.02 mg were used for all materials to avoid variations in thermal properties, being pressed into an aluminum cup to improve contact between the material and the heating furnace.

The temperature program started at holding for 1 min, then increased from 0 to 240 °C at a rate of 20 °C/min, followed by a 1 min hold at 240 °C.

#### Elemental Analysis

Elemental analysis was performed using an Elementar Vario MICRO cube apparatus (Langenselbold, Germany) in the CHNS mode. Organic chlorine analysis of lignin was conducted with a Bruker S4 Pioneer XRF spectrometer (USA) using a precalibrated MultiRes measurement method. For the analysis, lignins were mixed with NaHCO_3_ in a 1:10 ratio.

#### Thermogravimetry

TGA experiments on lignin and its derivatives were performed using a Netzsch STA 449F3 thermal analyzer (NETZSCH Instruments North America, Burlington, United States). Samples, weighing 5 ± 0.4 mg, were pyrolyzed in aluminum oxide crucibles under a nitrogen atmosphere at a flow rate of 40 mL/min. The pyrolysis experiments were conducted with a heating rate of 20 K/min, ranging from 20 to 380 °C.

#### Mechanical Testing

Test specimens obtained from the solvent casting (film) and 3D printing extruder process (filament) were mechanically tested with an Instron 5866 instrument (ASTM D638 standard) (Insron, US) and a load cell of 2.5 kN (force sensor capable of measuring up to 2.5 kN of force) used for tensile testing of the biocomposites. Five specimens of each series were tested. The speed and grip distances were 20 and 30 mm for a 10 mm wide film and 50 and 30 mm for extruded filament, respectively.

#### 3D-Printing Capability

The test specimen (bone-shaped) with dimensions of 30 × 4.96 × 0.94 mm was printed with the respective extruded materials using a Wanhao Duplicator 4s dual-extruder 3D printer (China) which was equipped with a 0.4 mm nozzle diameter. The print bed was tempered at 70 °C, and the extruder temperature was varied from 210 to 230 °C for all compositions. See Table S4 for the detailed parameters.

## Results and Discussion

### Characterization of Organosolv Pine Derivatives

We previously demonstrated the chloromethylation of aspen lignin^20^ and applied the same methodology to pine lignin. A new characteristic peak at 4.5–4.75 ppm in the NMR spectra confirms the presence of −CH_2_–Cl. Similarly, absorption peaks in the FT-IR spectra at 1413–142, 1264–1267, and 633–670 cm^–1^ indicate the presence of −CH_2_Cl groups. XRF analysis further confirms that 11.5% chlorine is present in the chloromethylated sample. These findings provide compelling evidence for the successful incorporation of chloromethyl groups into the lignin structure.

**Figure 1:**
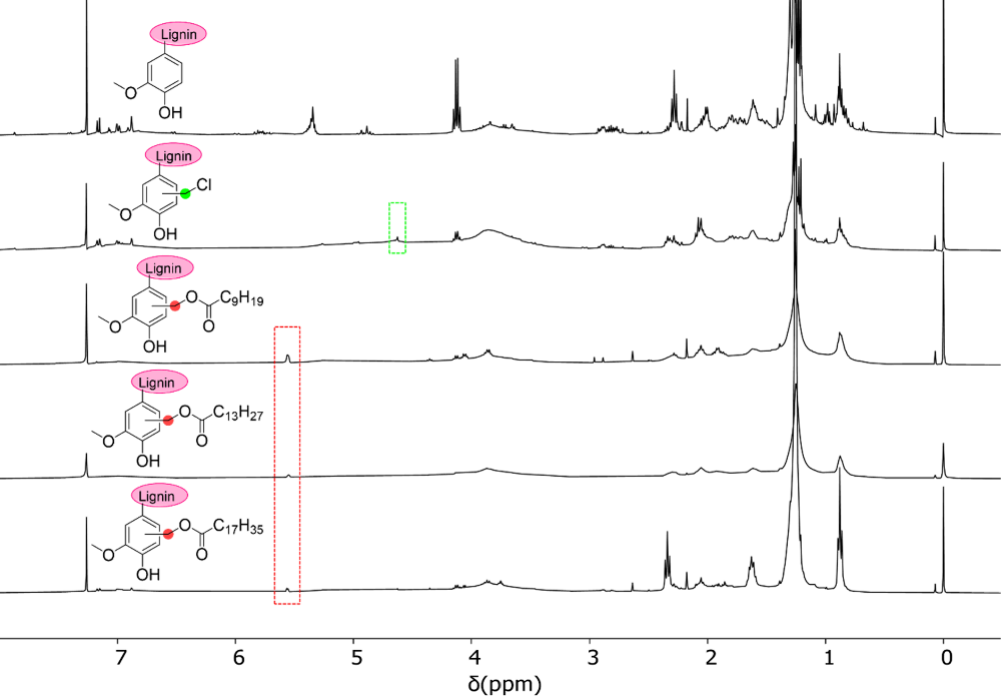
^1^H NMR spectra (from top to bottom) of organosolv pine lignin, chloromethylated lignin, and lignin esters in CDCl_3_.

The success of the esterification reaction was confirmed by ^1^H NMR studies. A comparison of the spectra of esterified lignin with the initial substrate, i.e., organosolv pine lignin, revealed the disappearance of the chloromethylated peak (ph–CH_2_–Cl) and the emergence of a new peak corresponding to the methylene moieties (ph–CH_2_–O) indicative of the esters, as illustrated in Figure 1.

**Figure 2:**
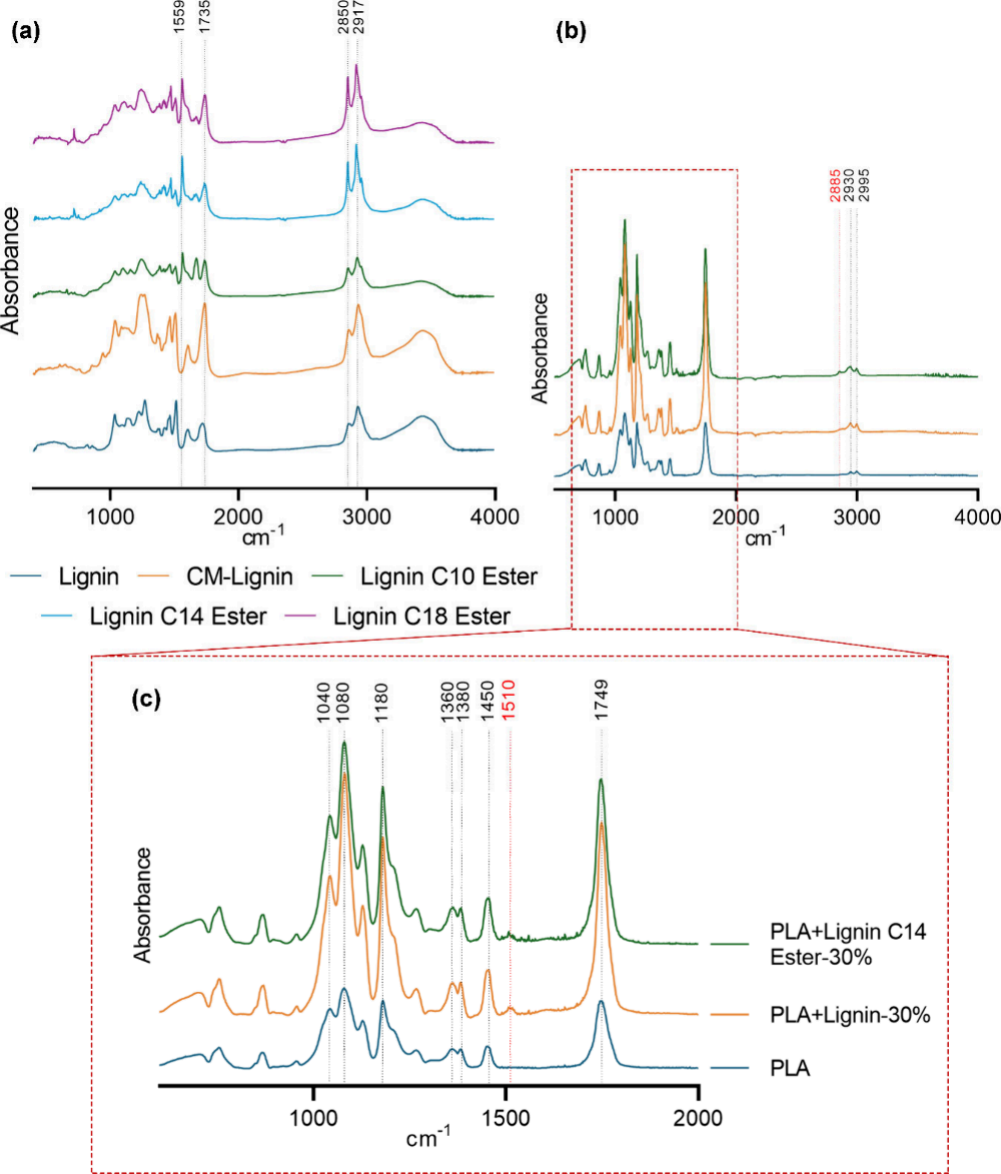
FT-IR spectra of organosolv pine lignin and its derivatives (a) and PLA/lignin blends (b,c).

The increased intensity of two bands around 2917 and 2850 cm^–1^ is due to CH_2_ stretching modes in the methyl and methylene groups of the ester side chains.^23^ Additionally, a major absorption band at 1735 cm^–1^ and bands at 1559 cm^–1^ correspond to C=O stretching vibrations and aromatic skeletal vibrations in lignin, respectively,^24^ as illustrated in Figure 2a. Lignin’s complex and diverse structure can cause considerable variation in the FT-IR spectrum, making it difficult to distinguish between peaks originating from native lignin and those resulting from esterification. In addition, elemental analysis (Table S2) shows that esterified lignin contains more C and H than native lignin. These changes strongly confirm the lignin esterification;^25^ PLA/lignin curves displayed bands similar to those of neat PLA curves (Figure 2b).

The asymmetric and symmetric stretching vibrations of the CH_3_ group were displayed in neat PLA at 2995 and 2930 cm^–1^, respectively. A C=O stretching vibration is responsible for the intense peak at 1749 cm^–1^. The peak at 1450 cm^–1^ can be attributed to CH_3_ antisymmetric bending vibrations. Peaks at 1380 and 1360 cm^–1^ correlate with CH group deformation and bending modes. C–O–C stretching vibrations are attributed to the peaks at 1180, 1080, and 1040 cm^–1^. Interestingly, this peak is notably higher in the PLA/lignin curve, indicating that the addition of lignin increased the hydroxy group content. In addition, biocomposites containing lignin showed a small peak at 1510 cm^–1^ caused by C=C groups in the aromatic rings and at 2885 cm^–1^ caused by CH_2_ stretching modes in the methyl and methylene groups of the ester side chain.

### Thermal Behavior: DSC and TGA of Esterified Lignin PLA Composite

DSC stands as one of the most widely accepted methods for determining the glass transition temperature of lignin molecules. This thermal parameter of polymers yields crucial insights into utilizing lignin in polymer applications and processing it via current industrial techniques such as hot pressing.

**Figure 3:**
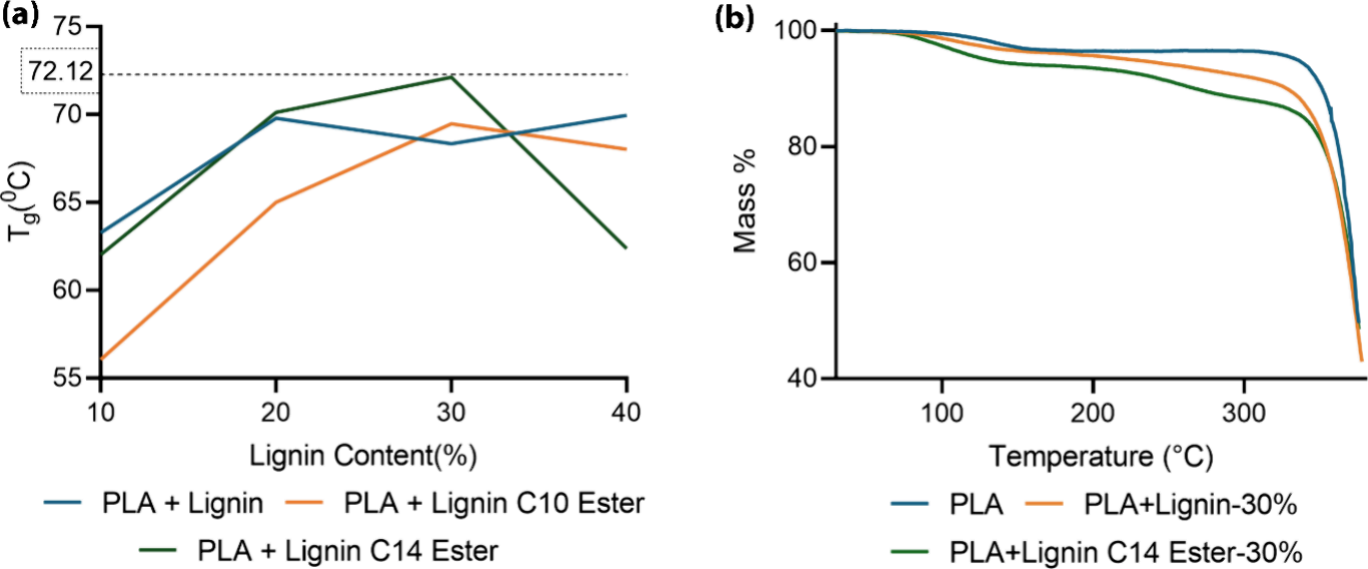
Glass transition temperature for PLA/lignin blends (a) and TGA plot of PLA and PLA/lignin composites obtained under a nitrogen atmosphere at 20 °C/min heating rate (b).

**Figure 4:**
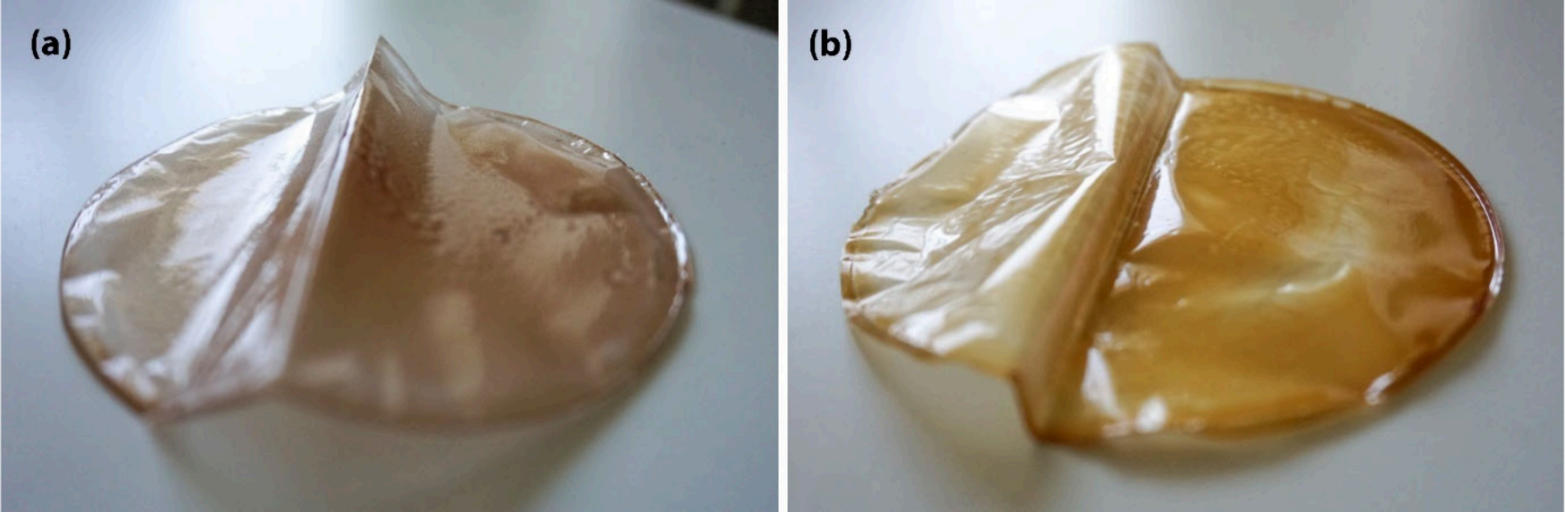
PLA + lignin-30% film (a) and PLA + lignin C14 ester-30% film (b).

Lignin is being explored as fillers, extenders, and reinforcing agents in rubber and thermoset resins,^26^ as well as serving as hard segments in the development of toughened thermally simulated shape-memory copolymeric elastomers.^27^ The initial study published on lignin modification with fatty acids demonstrated that esterified lignin acquired novel and intriguing properties, including alterations in solubility and thermal behavior,^28^ and a subsequent study noted a steady decline in *T*_g_ with the use of larger ester substituents.^29^ It is widely recognized that the reduction of *T*_g_ is more pronounced with longer attached chains. However, determining the *T*_g_ of lignins proves challenging due to the complexity of lignin chemistry and its broad molecular weight distributions, resulting in a typically wide temperature range for this phenomenon.^30^ Previous studies have addressed the thermoplasticity of lignin,^31^ suggesting that lignin molecules exhibit a thermal softening point.^32^ Typically, this transition occurs at elevated temperatures ranging from 90 to 180 °C for nonderivatized lignins.^33^ In this specific case, the *T*_g_ values for isolated pine lignins were determined as 111 and 68 °C for PLA. Nevertheless, PLA with lignin-ester derivatives exhibited a noteworthy alteration in their thermal characteristics. Lignin esters presented in this study show a good compatibility with PLA. Figure 3a and Table S3 illustrate the obtained thermograms for all PLA-lignin-ester derivatives. In the case of unmodified lignin samples, lignin esters with decanoic acid (C10) did not improve the *T*_g_. However, lignin esters with tetradecanoic acid (C14) at 30% gave the maximum *T*_g_ of 72.12 °C and dropped after further incorporation. The glass transition step in DSC curves is distorted in the case of stearic acid (C18), probably due to the heterogeneity of the material (Figure S1). Thus, the result of the present study suggests PLA + lignin C14 ester-30% to be chosen as the best candidate for further application. Photos of PLA + lignin-30% film and PLA + lignin C14 ester-30% film are given in Figure 4.

The thermogravimetry (TG) curves representing the weight loss percentage of PLA/lignin blends are shown in Figure 3b. These curves were obtained at a heating rate of 20 °C/min under a nitrogen atmosphere. The thermal degradation data indicate the rate of weight loss, which can be used to compare the thermal stability characteristics of different lignin materials. As illustrated in Figure 3b, thermal decomposition occurs over two distinct temperature ranges, beginning at approximately 80 and 245 °C. The weight loss at 80 °C is attributed to the evaporation of humidity and chemically bound water.^34^ The major decomposition starting at 245 °C marks the initial degradation temperature, with further degradation occurring at higher temperatures. This indicates that the PLA/lignin blends are thermally stable between 210 and 230 °C, making them suitable for 3D printing applications.

### Mechanical Testing

**Figure 5:**
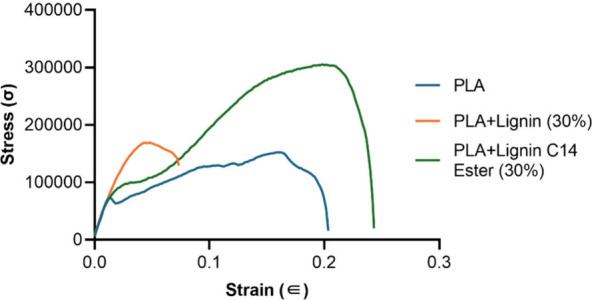
Experimental stress–strain curves for PLA, PLA + lignin-30% film, and PLA + lignin C14 ester-30% film.

Based on the tensile test results of PLA/Lignin film (Figures 4^5^ and 6a–c and Table S4), the addition of lignin to PLA affects its mechanical properties. The stress–strain plot (Figure 5) indicates that the mechanical properties of PLA can be significantly altered by blending it with lignin and its ester derivatives. Specifically, pure PLA shows moderate ductility and stress but does not achieve stress levels as high as those of the PLA-lignin ester blends. PLA + lignin (30%) results in lower strength and strain, suggesting that the material becomes more brittle with lignin addition. PLA + lignin C14 ester (30%) performs the best in terms of both strength and strain, reaching the highest stress and strain values, indicating increased toughness and strength. Overall, the addition of the lignin C14 ester to PLA provides the most beneficial impact on the mechanical properties, making it a promising candidate for applications requiring higher strength and toughness.

**Figure 6:**
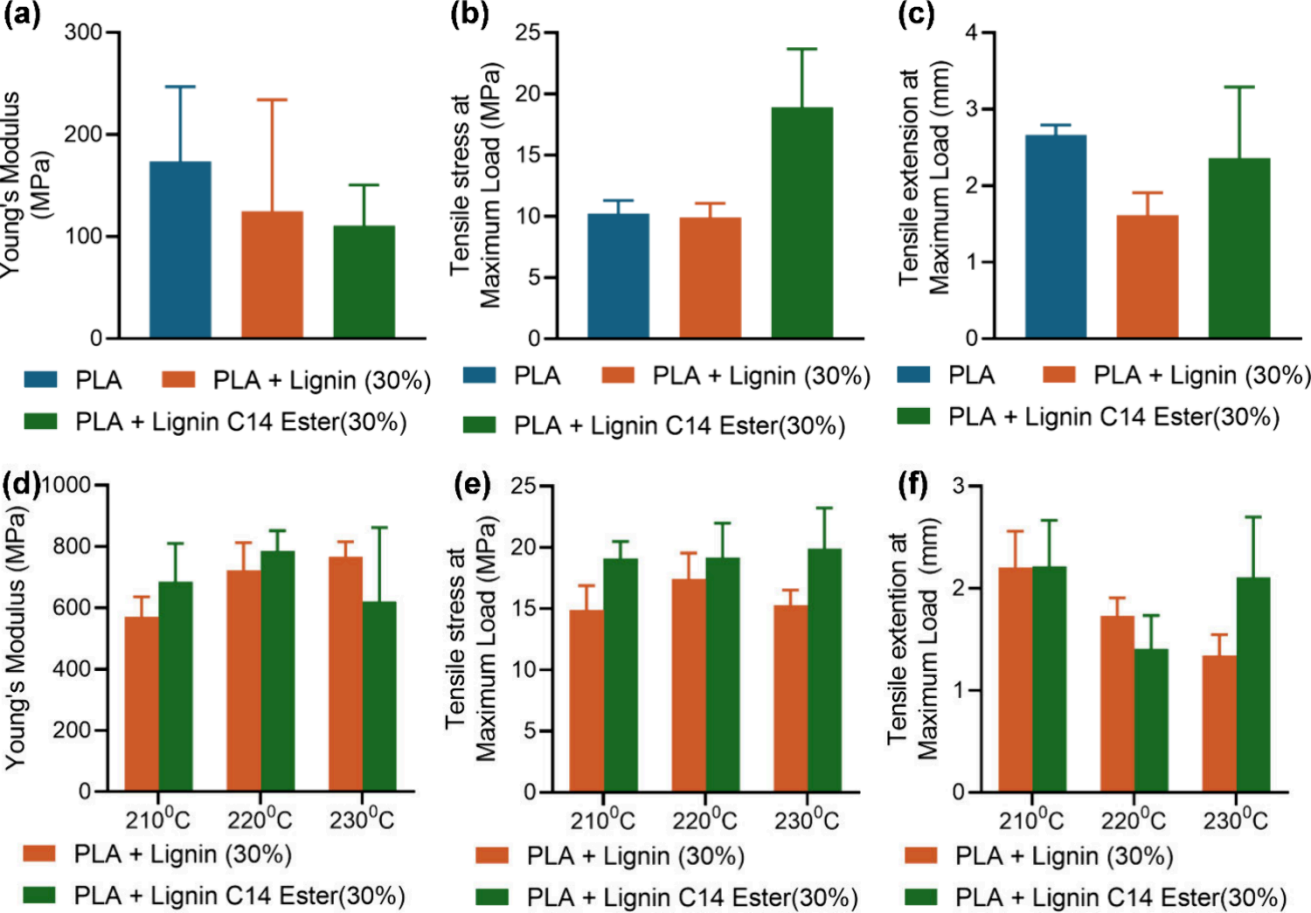
(a,d) Young’s modulus, (b,e) stress at maximum load, and (c,f) tensile extension at maximum load of PLA/lignin film (a–c) and PLA/lignin extruded filament (d–f).

The Young’s modulus (Figure 6a) of PLA/lignin films (Figure 4a) shows variation based on the lignin used. Incorporation of lignin tends to decrease the stiffness of the PLA matrix, with values generally lower than those of pure PLA. PLA + lignin C14–30% blend had the lowest modulus, indicating a reduction in stiffness. The PLA + lignin C14–30% blend exhibited the highest tensile stress at maximum load (Figure 6b) compared to both neat PLA and PLA + lignin-30% (Figure 4b), indicating that lignin ester can act as a filler that may strengthen the polymer matrix under stress. However, this blend showed a moderate tensile extension at maximum load, slightly lower than that of neat PLA but higher than that of PLA + lignin-30% (Figure 6c), suggesting that modified lignin is a more flexible material. These results suggest that while adding lignin to PLA through a solvent casting method reduces its stiffness; the effect is more pronounced with Lignin C14 ester.

The mechanical properties of the extruded filaments produced at various temperatures and compositions reveal distinct trends (Figure 6d–f and Table S5). Filaments with unmodified lignin and lignin C14 ester at different temperatures exhibited variations in tensile stress, tensile extension, and Young’s modulus. The addition of lignin C14 ester significantly improves the tensile stress at maximum load for filament specimens, making them more robust compared to neat material. This improvement is consistent across the different temperatures tested (Figure 6e). The tensile extension decreases with temperature but tends to be higher for filaments with lignin C14 ester at higher temperatures (Figure 6f). The Young’s modulus indicates that the blending process and lignin content significantly influence the stiffness. The Young’s modulus tends to increase with the processing temperature for both native lignin and lignin C14 ester samples, demonstrating peaks at 230 °C for lignin and 220 °C for lignin C14 ester (Figure 6d).

**Figure 7:**
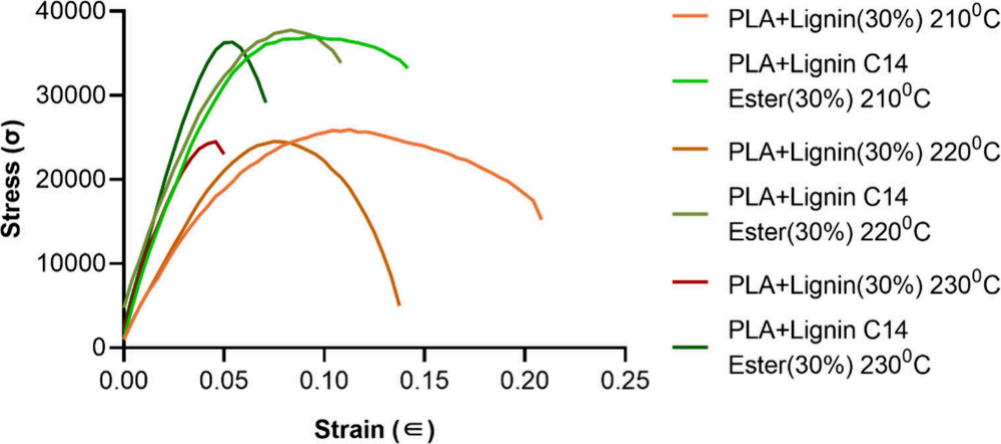
Experimental stress–strain curves for PLA and PLA + lignin filaments.

Similarly, the stress–strain curve (Figure 7) shows that PLA + lignin (30%) at 210 °C exhibits moderate tensile strength and a reasonable level of strain before fracture. This suggests that these materials have decent flexibility but lower strength compared to others. The PLA+lignin C14 ester (30%) displays the highest tensile strength and strain capability. These materials can endure higher stress and deformation before breaking, indicating that they are the strongest and most flexible in the data set. The filament preparation at 230 °C shows a material with lower tensile strength and a shorter strain range, meaning this material is more brittle, breaking earlier under stress for both lignin and lignin esters. Unmodified lignin with PLA is more brittle (lower strain before failure). However, lignin esters are more ductile, with higher strain values before failure, indicating better flexibility.

In conclusion, the incorporation of lignin into PLA significantly affects the mechanical properties of both films and extruded filaments, demonstrating increased mechanical properties in the composites with significantly higher lignin content compared to the literature.^10,35^ Our findings show that the addition of unmodified lignin slightly reduced the tensile stress and extension; however, lignin C14 ester significantly enhanced the tensile stress and extension at the break, suggesting improved compatibility and interaction within the PLA matrix. Lignin generally reduces the Young’s modulus of the PLA matrix and can potentially elasticize the polymer composite; it also imparts unique characteristics that might be advantageous for specific applications such as biodegradable packaging and low-load structural components. The findings underscore the importance of optimizing the content and type of lignin for achieving desirable mechanical properties in PLA/lignin composites, particularly for applications in 3D printing and other additive manufacturing technologies where material performance under mechanical stress is crucial.

### Morphological Characterization of 3D-Printed Specimens

**Figure 8:**
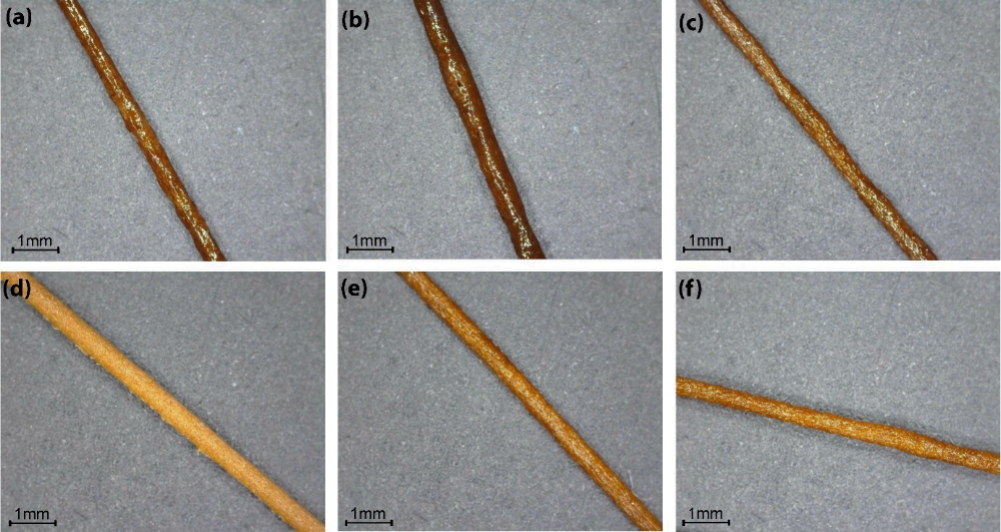
Individual filaments extruded from a 0.4 mm nozzle at 210 °C (a, d), 220 °C (b, e), and 230 °C (c, f) for PLA+ lignin-30% (a–c) and PLA + lignin C14 ester-30% (d–f).

Figure 8 shows individual filaments extruded from a 0.4 mm nozzle at different temperatures for PLA + lignin-30% and PLA + lignin C14 ester-30% composites. The comparison indicates that the addition of lignin C14 ester to PLA results in a filament with improved surface smoothness, especially at higher extrusion temperatures. The smoother surface morphology at 220 °C suggests enhanced compatibility and processing stability for the PLA + lignin C14 ester-30% composite, potentially leading to better mechanical properties and overall material performance.

**Figure 9:**
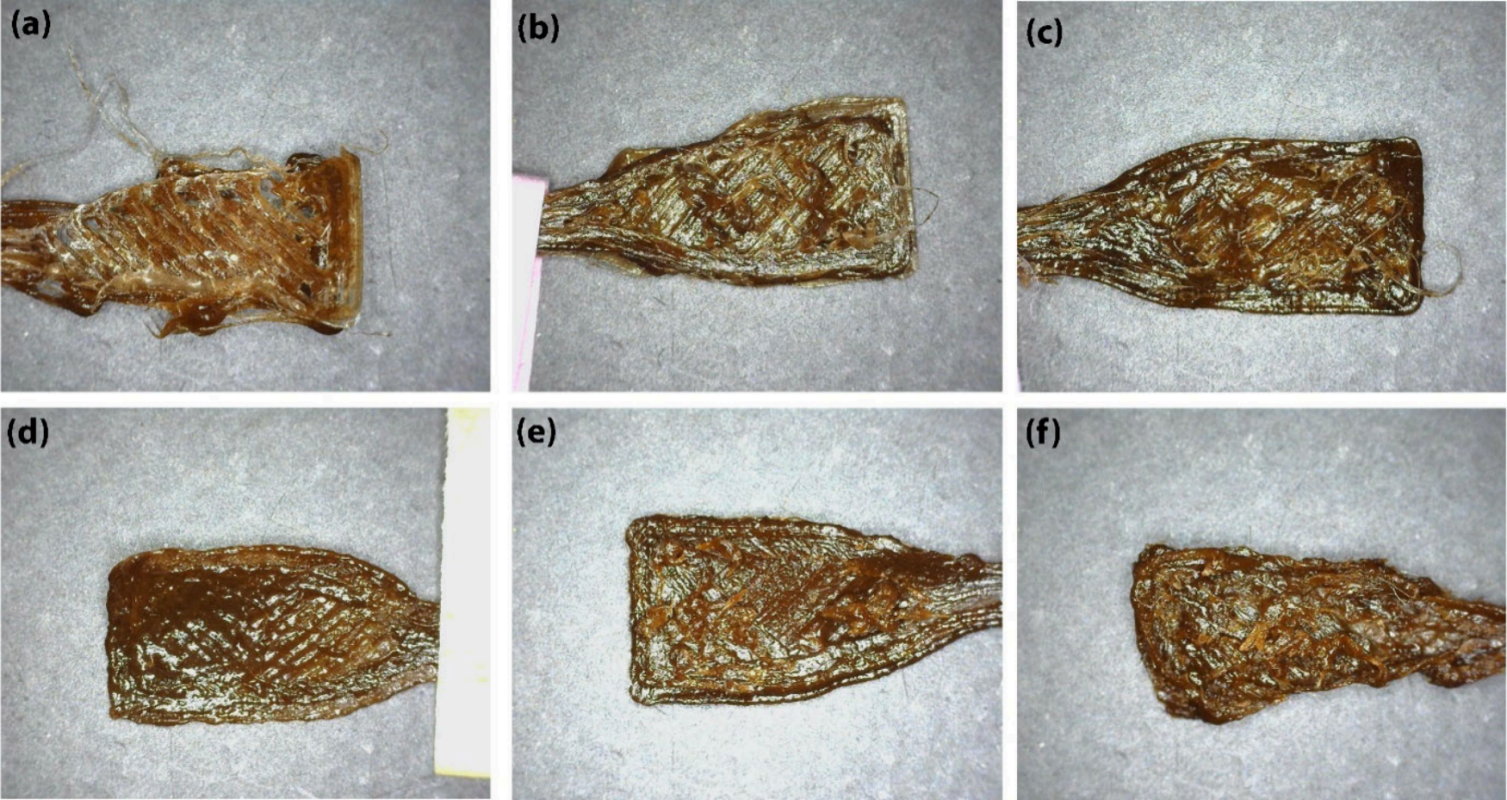
3D-printed bone-shaped samples at 210 °C (a, d), 220 °C (b, e), and 230 °C (c, f) for PLA + lignin-30% (a–c), and PLA + lignin C14 ester-30% (d–f).

Figure 9 shows the 3D-printed bone-shaped samples at different temperatures for PLA + lignin-30% and PLA + lignin C14 ester-30% composites. PLA + lignin-30% (Figure 9a–c) samples show significant structural irregularities and poor layer adhesion, indicating suboptimal printing conditions under all three printing conditions. However, PLA + lignin C14 ester-30% (Figure 9d–f) samples demonstrate better structural integrity compared to their PLA + Lignin-30% counterpart at the same temperature but with some visible defects. At 220 °C (Figure 9e) the sample displays the highest quality among all, with a uniform surface texture and strong layer cohesion, indicating optimal printing conditions. The comparison suggests that the incorporation of lignin C14 ester into PLA results in better 3D printability, especially at higher extrusion temperatures. The improved layer adhesion and structural integrity of PLA + lignin C14 ester-30% composites at 220 °C highlight the enhanced compatibility and stability of this material combination, making it more suitable for 3D printing applications compared to PLA + lignin-30%.

## Conclusions

Organosolv pine lignin was modified through chloromethylation, followed by esterification with decanoic acid (C10), tetradecanoic acid (C14), and stearic acid (C18), and subsequently characterized. Each lignin ester was blended with PLA at four different percentages (10%, 20%, 30%, and 40%) using the solvent casting technique. The blended polymers were subjected to DSC analysis, revealing that the introduction of ester moieties into lignin affected the thermal properties of the polymers. PLA could be filled up to 30% of lignin esters, which results in a slight increase in the *T*_g_ of a composite. PLA/lignin-ester composites were found to be suitable for 3D printing applications. Lignin-esters are compatible with PLA and this increases their thermal stability and mechanical properties, which could be used for the development of high-performance bioplastics with extended working temperature ranges.

Thermogravimetric analyses indicated that the PLA/lignin blends are thermally stable between 210 and 230 °C, with degradation temperatures starting at 245 °C. The addition of lignin and esterified lignin to PLA significantly influenced the mechanical properties of both films and extruded filaments. A comparison of 3D-printed specimens suggests that incorporating lignin C14 ester into PLA results in better 3D printability, especially at higher extrusion temperatures. The improved layer adhesion and structural integrity of PLA + lignin C14 ester-30% composites at 220 °C highlight the enhanced compatibility and stability of this material combination, making it more suitable for 3D printing applications compared to PLA + lignin-30%. The unusual behavior of C14 being both the most flexible and the strongest compared to C10 and C18 carbon chain esters with PLA can be attributed to the optimal balance between chain length and molecular interactions. In the case of C14, the carbon chain is long enough to provide flexibility through increased chain mobility but not so long as to cause excessive chain entanglement or phase separation, which could compromise the strength. In contrast, shorter chains such as C10 may not offer sufficient flexibility, and longer chains such as C18 can lead to more rigid structures due to stronger intermolecular forces or crystallization, reducing overall flexibility. C14 strikes a balance between these effects, leading to an improved flexibility and strength in the composite. This balance between flexibility and strength is less commonly observed but can be explained by the unique interplay of chain length, mobility, and intermolecular interactions specific to this system. Successful incorporation of lignin-based fillers could reduce the price of a bioplastic composite and 3D printing with it. Esterification of lignin is proven to be a greener and more sustainable approach for 3D printing materials development. This strategy to transform biomass into building material gradually reduces greenhouse gas emissions from the building sectors and ensures sustainability, aligning with the United Nations Sustainable Development Goals.

## Supporting Information Available

PLA/lignin combinations, elemental composition of lignin esters, DSC values for lignin PLA composites, thermal properties of studied lignin products, mechanical properties of PLA/lignin film, mechanical properties of PLA/lignin extruded filament, and filament fabrication toolpath and process parameters (PDF)

## Abbreviations

AcOH: acetic acid
CML: chloromethylated lignin
CHO: formaldehyde
HCl: hydrochloric acid
DMSO: dimethyl sulfoxide
EA: elemental analysis
EtOH: ethanol
FT-IR: Fourier-transform infrared spectroscopy
NMR: nuclear magnetic resonance
PFA: paraformaldehyde
XRF: X-ray fluorescence
