# Supplementary material for: Novel Softwood Lignin Esters as Advanced Filler to PLA for 3D Printing
Source: ACS Omega. 2024 Oct 24;9(44):44559–67. doi: 10.1021/acsomega.4c06680 (PMC11541478; doi:10.1021/acsomega.4c06680)
Supplement: Supplementary file 1 — ao4c06680_si_001.pdf [file ao4c06680_si_001.pdf]

ELECTRONIC SUPPORTING INFORMATION

Novel Softwood Lignin Esters as Advanced Filler to PLA for 3D Printing

Mahendra K. Mohan†, *Illia Krasnou*‡, *Tiit Lukk*†, *Yevgen Karpichev**†

†Department of Chemistry and Biotechnology, Tallinn University of Technology (TalTech), Akadeemia tee 15, 12618 Tallinn, Estonia

‡Department of Materials and Environmental Technology, Tallinn University of Technology (TalTech), Ehitajate tee 5, 19086, Tallinn, Estonia

Corresponding Author:

*Dr. Yevgen Karpichev, fax: +372 620 2994

E-mail: yevgen.karpichev@taltech.ee

Table S1. Overview of PLA/Lignin combinations

Blend	Lignin (%)	Blend	Lignin (%)
PLA	0	PLA/Lignin_C14	10
PLA/Lignin	10	PLA/Lignin_C14	20
PLA/Lignin	20	PLA/Lignin_C14	30
PLA/Lignin	30	PLA/Lignin_C14	40
PLA/Lignin	40	PLA/Lignin_C18	10
PLA/Lignin_C10	10	PLA/Lignin_C18	20
PLA/Lignin_C10	20	PLA/Lignin_C18	30
PLA/Lignin_C10	30	PLA/Lignin_C18	40
PLA/Lignin_C10	40		

Table S2. Elemental composition of Lignin esters.

Sample	N%	C%	H%
SMP	n.d.	61.79	6.24
CMP	n.d.	61.63	7.04
C10	n.d.	62.3	8.3
C14	n.d.	66.6	8.4
C18	n.d.	67.3	9

Table S3. DSC properties of Lignin PLA composites

Sample	T_g	T_m
PLA	68.66	151.66
PLA + Lignin-10%	63.28	149.02
PLA + Lignin-20%	69.79	149.65
PLA + Lignin-30%	68.35	148.99
PLA + Lignin-40%	69.96	148.03
PLA + Lignin C10 Ester-10%	56.06	151.34
PLA + Lignin C10 Ester-20%	65	150.36
PLA + Lignin C10 Ester-30%	69.48	148.66
PLA + Lignin C10 Ester-40%	68.02	149.36
PLA + Lignin C14 Ester-10%	62.03	147.65
PLA + Lignin C14 Ester-20%	70.12	148.99
PLA + Lignin C14 Ester-30%	72.12	150.34
PLA + Lignin C14 Ester-40%	62.38	149.36
PLA + Lignin C18 Ester-10%	---	149.68
PLA + Lignin C18 Ester-20%	---	147
PLA + Lignin C18 Ester-30%	---	149.33
PLA + Lignin C18 Ester-40%	---	147.64

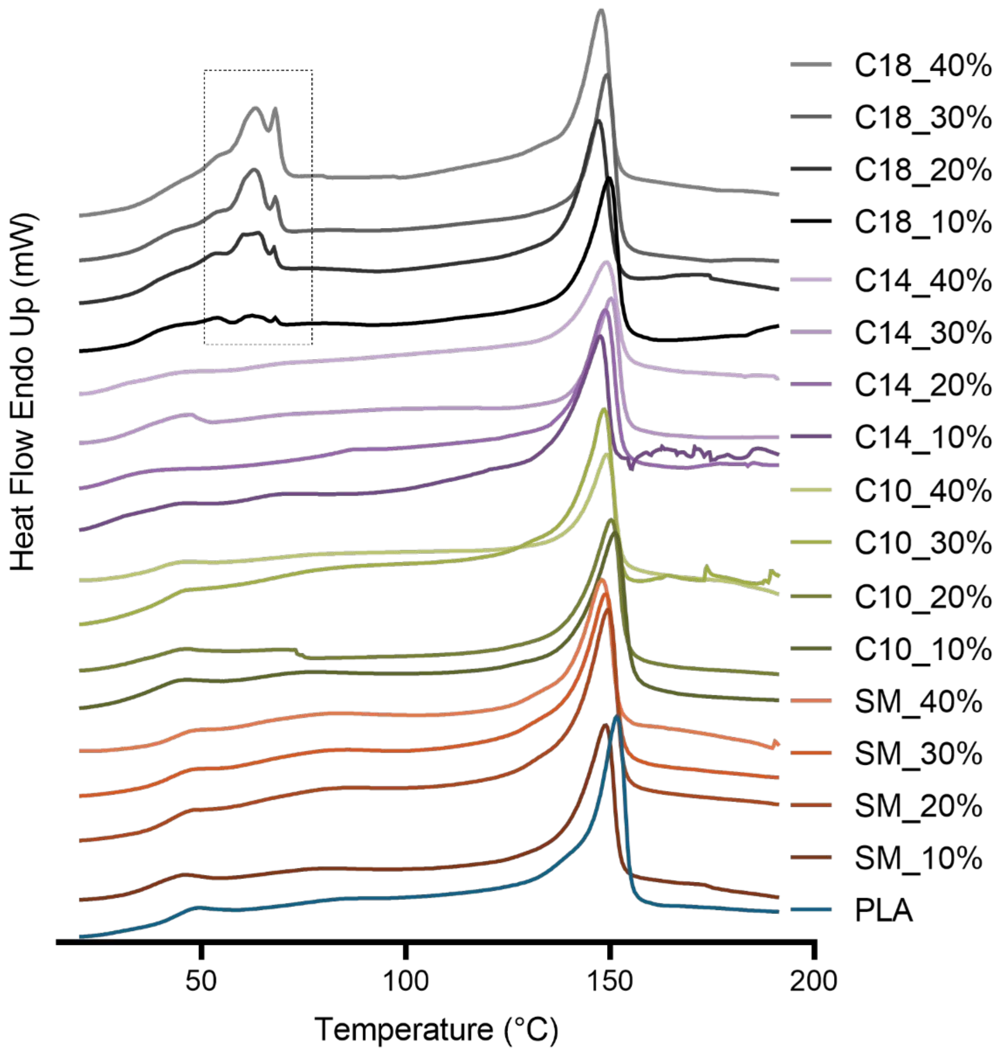

Figure S1. Thermal properties of studied lignin products

Table S4. Mechanical properties of PLA/lignin film

Specimen	Maximum Load (N)	Tensile stress at Maximum Load (MPa)	Tensile extension at Maximum Load (mm)	Modulus of Elasticity	Width (mm)	Thickness (mm)
PLA film	4.6±0.3	10.2±1	2.6±0.13	173.5±73	10	0.04±0.005
PLA + Pine (30%)	5.1±0.67	9.9±1.2	1.6±0.3	124.8±109.3	10	0.05
PLA + C14 (30%)	5.5±1	18.9±4.7	2.3±0.9	110.5±40	10	0.028±0.01

Table S5. Mechanical properties of PLA/lignin extruded filament

Specimen	Modulus of Elasticity	Maximum Load (N)	Tensile stress at Maximum Load (MPa)	Tensile strain at Maximum Load (%)
SM-210℃	571.5±64.5	4.6±0.3	14.9±2	7.14±1.2
SM-220℃	723±90.2	4.2±0.3	17.5±2.1	5.6±0.6
SM-230℃	766.1±45.5	3.88±0.3	15.3±1.2	4.4±0.7
C14-210℃	685.7±124.4	5±0.6	19.1±1.4	7.2±1.5
C14-220℃	786.14±66	4±0.2	19.2±2.8	4.52±1
C14-230℃	620.44±241.6	4.33±0.39	19.93±3.3	6.72±1.93

Table S6. Filament fabrication toolpath and process parameters

Toolpath parameters				Process parameters	
Nozzle diameter	0.40mm	Printing speed	900mm/min	Extruder temperature	210-230^{0}C
Extrusion multiplier	0.90	Speed for non-print moves	3600mm/min	Extrusion multiplier	1
Extrusion width	0.40mm	Layer height	0.1mm	Building platform heating	70^{0}C
Infill density	20%	Infill pattern	Rectilinear	Fan speed (%)	60%
Extrusion width	Equal to nozzle diameter	First layer speed	10%	Dog bone dimension	X- 30mm<br>Y- 4.96mm<br>Z- 0.94mm
